# A Single-Centre Experience of First-Line Romiplostim and Immunosuppressive Therapy in Patients With Aplastic Anemia

**DOI:** 10.7759/cureus.37682

**Published:** 2023-04-17

**Authors:** Gaurav Dhingra, Ashok Rajoreya

**Affiliations:** 1 Department of Clinical Hematology, All India Institute of Medical Sciences, Rishikesh, Rishikesh, IND; 2 Hematology Hemato-Oncology Center, Ratan Jyoti Netralaya Apollo Spectra Hospitals, Gwalior, IND

**Keywords:** immunosuppressant therapy, cyclosporine (csa), anti-thymocyte globulin (atg), romiplostim, aplastic anemia

## Abstract

Background

Romiplostim, a thrombopoietin (TPO) receptor antagonist, promotes tri-lineage hematopoiesis in patients with acquired aplastic anemia (AA). However, its efficacy as a first-line treatment in combination with an immunosuppressant, i.e., anti-thymocyte globulin (ATG) and cyclosporine (CSA), remains unexplored.

Objective

To assess the efficacy and safety of romiplostim in combination with ATG and CSA as first-line treatment in patients with AA.

Method

A single-center, retrospective study of AA patients, where data of patients administered with ATG + CSA + romiplostim as a first-line treatment was included. Romiplostim 5 µg/kg weekly for one month; post that, the dose was increased to 10 µg/kg weekly for the next five months. The primary outcome involves the overall response rate and hematological response at baseline, three months, and six months.

Result

Data from 12 patients with a median age of 18 years was evaluated. At a median follow-up of six months, 25% achieved a complete response, 41.6% achieved a partial response, and 16.7% had no response. Improvement in tri-lineage hematopoietic response had been seen at six months from baseline, with improvement in absolute neutrophil count (ANC) and platelet count (PC) being the most significant, with an increase of >100% from baseline, followed by total leukocyte count (TLC) (75.13%) and hemoglobin (Hb) (66.07%) from baseline. Two deaths were reported during the treatment.

Conclusion

Romiplostim, in combination with ATG plus CSA, demonstrated clinically significant outcomes as a first-line treatment in patients with AA. Further studies are required to confirm these findings in larger populations to assess long-term outcomes.

## Introduction

Acquired aplastic anemia (AA) is an immune-mediated hematopoietic disorder characterized by pancytopenia and hypocellular bone marrow [1]. The disease results in the activation of cellular pathways that lead to the destruction of hematopoietic stem cells and progenitor cells. At the cellular level, CD8+ and CD28- cytotoxic T-helper cells are activated, reducing cell division and promoting hematopoietic stem cell apoptosis [2]. AA is a rare, life-threatening disease with a reported incidence of 1.6 cases/1,000,000/year [3]. AA patients are prone to severe infections due to neutropenia, experience bleeding due to thrombocytopenia, and/or fatigue due to anemia [4,5]. AA is associated with higher mortality, especially when pancytopenia becomes severe. Diagnosis of AA requires a bone marrow biopsy, and an accurate diagnosis and timely management are needed to prevent morbidity and mortality of the patients [6]. Treatment options for AA depend on the severity of the condition and may include observation, blood transfusions, medications, or bone marrow transplantation [7]. Since the disease includes the failure of the bone marrow cell, the gold standard for diagnosis of AA involves bone marrow examinations. In severe AA cases, bone marrow transplantation or hematopoietic stem cell transplantation (HSCT) is recommended. However, the patients who are not eligible for bone marrow transplantation, immunosuppressant therapy (IST) is the cornerstone treatment involving anti-thymocyte globulin (ATG) and cyclosporine (CSA) [8]. The literature survey suggests that 30% of the patients do not respond to the IST and 30-40% of patients that respond relapse eventually [9,10]. However, 30-70% of relapsed patients responded to the second line of IST or CSA alone [10,11]. Other non-transplant therapeutic options include alemtuzumab, danazol, and cyclophosphamide, which give unsuccessful responses [12]. For severe AA patients who were non-responsive or had an insufficient response to IST, supportive care (e.g., blood transfusion) was the prime treatment option until US FDA approved the oral thrombopoietin (TPO) receptor agonist eltrombopag in combination with IST in 2014 as the first-line treatment for refractory AA. Eltrombopag showed a trilineage response in AA refractory to IST [13]. TPO is the key factor that binds to the TPO receptor on megakaryocytes and drives the production of platelets. The present study deals with another TPO receptor agonist, Romiplostim, a peptide body that stimulates endogenous TPO production. Romiplostim is an Fc-peptide fusion protein (peptide) that signals and activates intracellular transcriptional pathways via the TPO receptor [14]. The possible mechanism of hematopoietic recovery by romiplostim is the stimulation of hematopoietic stem and progenitor cells (HSPCs), as the TPO receptor is expressed on HSPCs [15]. Romiplostim binds to and activates the TPO receptor on megakaryocyte precursors, promoting cell proliferation and viability and increasing platelet production. Romiplostim has already been approved in various countries for treating refractory immune thrombocytopenia [16]; therefore, its efficacy in AA needs to be explored. The present study reported the safety and efficacy of Romiplostim in combination with IST (ATG plus cyclosporin) as a first-line treatment for AA patients in RJN Apollo Spectra Hospitals, located in Gwalior, Madhya Pradesh.

## Materials and methods

Study design and participants

This is a single-center retrospective study conducted at Ratan Jyoti Netralaya Apollo Spectra Hospitals, located in Gwalior, Madhya Pradesh, between the time period from June 2021 to September 2022. The institutional ethical committee approved this study and granted a waiver of informed consent for the retrospective conduction of the study using the existing data. A total of 12 AA patients data, aged eight years or older, were included in the study. All the patients included have confirmed diagnosis of AA through bone marrow and cytogenetic studies, thrombocytopenia (i.e., platelet count ≤30 × 10⁹/L), and an Eastern Cooperative Oncology Group (ECOG) performance status score of 2 or lower. 

Procedure

Eligible patients were given Romiplostim 5 µg/kg weekly for one month. The dose was increased to 10 µg weekly for the next five months. The protocol for IST included: ATG equine was administered at a dose of 40 mg/kg/day for four days, along with CSA at a dose of 5 mg/kg/day for 12 months, and then tapered by 25 mg every three months as per guidelines.

Outcome

The primary endpoint was the overall response rate (ORR) in terms of complete response rate (CRR) and partial response rate (PRR) at baseline, three months (12 weeks), and six months (24 weeks) of therapy. At each time point, hemoglobin level (Hb), platelet count (PC), absolute neutrophil count (ANC), and total leukocyte count (TLC) were calculated to define CRR and PRR.

Safety

All the adverse events (AEs) reported during the study were graded by Common Terminology Criteria for Adverse Events (CTCAE) v4.0.

Statistical analysis

The data collected were pooled in a Microsoft Excel spreadsheet and then transferred for statistical calculations to the SPSS (version 21) software. The primary endpoint was reported with two-sided 95% CIs, calculated using paired t-test. A p-value of <0.05 is considered statistically significant.

## Results

Efficacy

Data from 12 AA patients were included in the study. The baseline demographics have been listed in Table 1.

**Table 1:** Baseline demographic and clinical characteristics of the study population (N=12). AA: Aplastic anemia.

Characteristics	Value
Males/Female, n (%)	3/9 (25/75)
Median Age (Range)	18 years (8-65 years)
Presenting symptoms	
Anemia, n (%)	12 (100%)
Infections, n (%)	9 (75%)
Bleeding sites, n (%)	
Gum	5 (41.66%)
Skin	4 (33.33%)
Ocular	2 (16.66%)
Per vaginal	4 (33.33%)
Severity of AA	
Non severe, n (%)	1 (8.3%)
Severe, n (%)	8 (66.66%)
Very severe, n (%)	3 (25%)
Laboratory parameters	
Total patient (N), Mean Hemoglobin (mg/dl), (SD)	12, 5.5 (1.77)
Total patient (N), Median platelet count (count/cu.mm), (SD)	12, 8875 (3451)
Total patient (N), Mean ANC (count/cu.mm), (SD)	10, 500.8 (276)
Total patient (N), Total Leucocyte count (count/cu.mm), (SD)	11, 2271.6 (1151)

The portion of patients, who received any hematological response at 12 weeks and 24 weeks, was 50% and 66.67%, respectively. At three months of follow-up, compared to baseline, 8.3% achieved a complete response, 41.60% achieved a partial response, and 33.33% showed no response, whereas at six months of follow-up, compared to baseline, 25% achieved a complete response, 41.6% patients achieved a partial response, and 16.7% had no response (Table 2).

**Table 2:** Overall response rate during the ATG + CSA and romiplostim treatment. ATG: Anti-thymocyte globulin; CSA: Cyclosporine.

		Treatment span (N=12)	
		3 months	6 months
Overall Response Rate (ORR)	Complete Response Rate (CRR)	1 (8.3%)	3 (25%)
	Partial Response Rate (PRR)	5 (41.60%)	5 (41.60%)
Total		6 (50%)	8 (66.67%)
No Response		4 (33.33%)	2 (16.7%)

**Figure 1:**
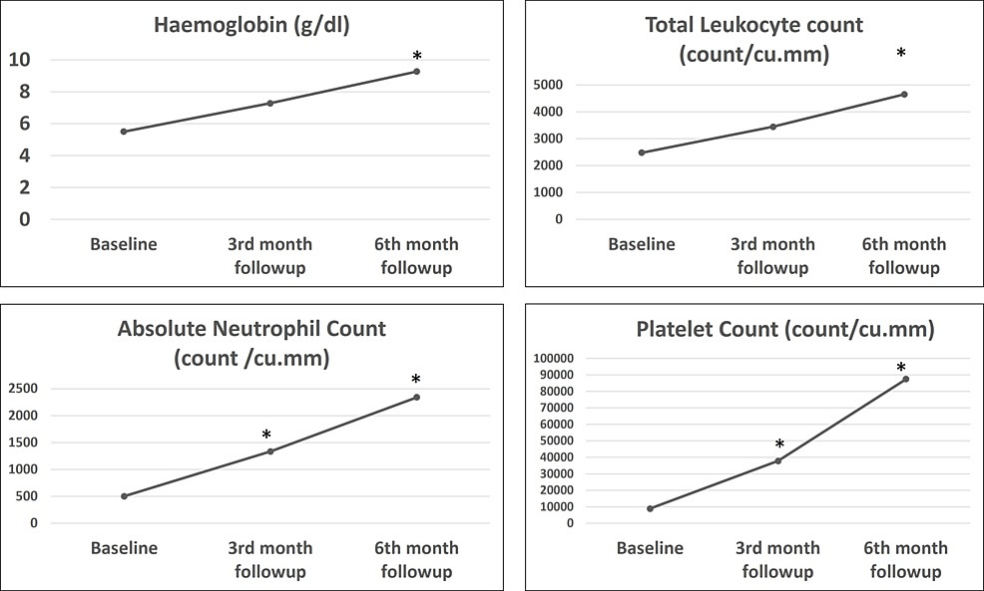
Tri-lineage response with ATG + CSA and romiplostim. * p-value < 0.05 was statistically significant. ATG: Anti-thymocyte globulin; CSA: Cyclosporine.

Improvement in trilineage hematopoietic response has been seen at six months from baseline, with improvement in ANC and PC being the most significant, with an increase of >100% from baseline, followed by TLC (75.13%) and Hb (66.07%) from baseline. Hemoglobin level and TLC showed significant improvement at the end of the study (six months). In contrast, ANC and PC showed statistically significant improvement at both three months and six months (Table 3 and Figure 1).

**Table 3:** Mean values of Hb, TLC, ANC, and platelet count at baseline, three months, and six months. Note: * p-value < 0.05 was statistically significant. cmm: Cells per cubic millimeter.

	Baseline values (mean±SD)	Values at 3 months (mean±SD)	P-value	Values at 6 months (mean±SD)	P-value
Haemoglobin (Hb) g/dl	5.6±1.9	7.3±1.9	0.169	9.3±2	0.010*
Total leukocyte count (TLC) cmm	2662.2±999.5	3544.4±1497.6	0.216	4662.5±1611.5	0.048*
Absolute neutrophil count (ANC) cmm	501±251.7	1443.8±603.2	0.007*	2435.7±916.8	0.001*
Platelet count (PC) cmm	8300±3497.6	37800±25507.3	0.006*	87444.4±60145.5	0.005*

Safety

There were two treatment-related deaths within three months of follow-ups, one with sepsis involving pneumonia and another with cerebral hematoma with infection. The frequently reported AEs were febrile neutropenia (25%), hypertension (8.33%), gum hypertrophy (8.33%), intracranial hemorrhage (8.33%), posterior reversible encephalopathy syndrome (PRES) (8.33%), and serum sickness (8.33%) (Table 4).

**Table 4:** Adverse event during the ATG + CSA and romiplostim treatment. ATG: Anti-thymocyte globulin; CSA: Cyclosporine; PRES: Posterior reversible encephalopathy syndrome; AE: Adverse event.

S. No.	Adverse Events	No. of Patients (N=12)
1	Treatment-related AEs (TRAEs)	8 (66.66%)
2	Death	2 (16.67%)
3	Febrile neutropenia	3 (25%)
4	Hypertension	1 (8.33%)
5	Gum hypertrophy	1 (8.33%)
6	Intracranial hemorrhage	1 (8.33%)
7	PRES	1 (8.33%)
8	Serum Sickness (Grade 2)	1 (8.33%)

## Discussion

In the present study, we have assessed the portion of patients achieving the hematological responses to romiplostim, a c-MPL agonist, with IST at two different dosages in patients with AA. Based on the current literature, we started with a lower dose of 5 ug/kg/week for one month and slowly increased to 10ug/kg/week for six months [17]. The primary endpoint was the ORR and the hematological response, and the dose modification led to significant improvement in ORR and hematological response in AA patients. The literature review suggested that the efficacy of the historic combination of ATG plus CSA in 42 patients of severe AA showed an ORR of 50% (24/42) at three months and 62% (26/42) at six months [18]. Compared to this, the present study of romiplostim and IST demonstrates an ORR of 50% at week 12 and a higher ORR of 66.67% at 24 weeks. The ORR of romiplostim and IST (ATG plus CSA) is higher than the historic combination of ATG plus CSA alone. Furthermore, the combination of eltrombopag and immunosuppressant therapy showed an ORR of 68% at the end of 24 weeks, which was similar to the present combination [19]. A similar study that analyzed the efficacy of romiplostim and IST (ATG plus CSA) in AA patients has been conducted in Japan. A total of 26 patients were screened, of which 17 AA patients (five transfusion-dependent non-severe AA, six severe AA [SAA], and six very SAA) were enrolled in the study, and the median age was 44.0 years (range: 25-70). Out of the 17, two patients discontinued romiplostim before week 27. The ORR at week 27 was 76.5% (95% CI: 50.10%, 93.13%), of which six (35.3%) achieved CR. The ORR at week 14 was 41.2% (95% CI: 18.44%, 67.03%). For patients who were dependent on platelet transfusion before romiplostim administration, 87.5% achieved transfusion independence or showed a reduction of transfusion requirement at week 27. In addition, to this, of those patients who required erythrocyte transfusion at baseline, 81.3% of them achieved transfusion independence or showed a reduction of transfusion requirement at week 27. The frequently reported AEs were constipation (41.2%) and headache (35.6%). The frequently reported drug-related AEs were headache (12.9%) and muscle spasms (9.7%) [20]. This regimen produced higher ORR at week 27 (76.5%) than those of historical control who received rabbit ATG plus CSA (approximately 50% at six months) [18].

In the present study, we used the same combination for different ethnicity. The Indian population is considered in the group of South Asian race. Compared to the Japanese population, the combination demonstrated low ORR. However, overall, the combination showed a significant clinical response with an acceptable toxicity profile and may serve as a new first-line treatment option in patients with AA.

Limitations

The major limitation of the present study was the recruitment of a relatively small number of patients (N=12) and the inclusion of a heterogeneous patient population (varying age) with respect to their disease severity. However, the recruitment of a heterogenous population is justified given the condition’s rarity. Another limitation is that the study did not test for hereditary AA at screening, which might have affected the results. Also, long-term follow-up data will be required to confirm the durability of the response and monitor for potential clonal evolution. Lastly, it is a retrospective study; therefore, all the limitations of a retrospective study also apply to this study.

## Conclusions

Romiplostim is known for its effectiveness in treating thrombocytopenia. The present study is a classic example where romiplostim, in combination with IST, appears to be effective and well-tolerated in patients with AA. Therefore, it can be considered a new addition to the armamentarium to treat AA.
